# Construction of cascade circuits for dynamic temporal regulation and its application to PHB production

**DOI:** 10.1186/s13068-023-02416-x

**Published:** 2023-10-27

**Authors:** Xiaomeng Li, Qingsheng Qi, Quanfeng Liang

**Affiliations:** 1 State Key Laboratory of Microbial Technology, Shandong University, Qingdao, 266237 People’s Republic of China; 2 The Second Laboratory of Lanzhou Institute of Biological Products Co., Ltd, Lanzhou, 730046 People’s Republic of China

**Keywords:** Quorum sensing, Cascade circuit, Temporal expression, Dynamic regulation, Poly-β-hydroxybutyric acid

## Abstract

**Background:**

To maximize the production capacity and yield of microbial cell factories, metabolic pathways are generally modified with dynamic regulatory strategies, which can effectively solve the problems of low biological yield, growth retardation and metabolic imbalance. However, the strategy of dynamic regulating multiple genes in different time and order is still not effectively solved. Based on the quorum-sensing (QS) system and the principle of cascade regulation, we studied the sequence and time interval of gene expression in metabolic pathways.

**Results:**

We designed and constructed a self-induced dynamic temporal regulatory cascade circuit in *Escherichia coli* using the QS system and dual regulatory protein cascade and found that the time intervals of the cascade circuits based on the Tra, Las system and the Lux, Tra system reached 200 min and 150 min, respectively. Furthermore, a dynamic temporal regulatory cascade circuit library with time intervals ranging from 110 to 310 min was obtained based on this circuit using promoter engineering and ribosome binding site replacement, which can provide more selective synthetic biology universal components for metabolic applications. Finally, poly-β-hydroxybutyric acid (PHB) production was taken as an example to demonstrate the performance of the cascade circuit library. The content of PHB increased 1.5-fold. Moreover, circuits with different time intervals and different expression orders were found to have different potentials for application in PHB production, and the preferred time-interval circuit strain C2-max was identified by screening.

**Conclusions:**

The self-induced dynamic temporal regulation cascade circuit library can enable the expression of target genes with sequential changes at different times, effectively solving the balance problem between cell growth and product synthesis in two-stage fermentation and expanding the application of dynamic regulatory strategies in the field of metabolic engineering.

**Supplementary Information:**

The online version contains supplementary material available at 10.1186/s13068-023-02416-x.

## Background

Metabolic engineering is a purposeful and rational modification technique that genetically manipulates chemical reactions catalyzed by intracellular enzymes, thereby maximizing the yield and productivity of target compounds [1]. Among them, the dynamic regulation strategy can effectively modify metabolic pathways and solve the problems of low biological yield, growth retardation and metabolic imbalance [2, 3]. However, the dynamic regulatory elements at this stage have the limitations of a small number of types, narrow response thresholds, limited regulatory ranges and unstable regulatory times and are mainly applied to regulate the expression of single genes at specific time points. Therefore, there is a need to expand the application scope of dynamic regulation strategies and explore novel synthetic biology generic components for metabolic engineering optimization to provide certain new ideas for solving the balance between cell growth and product synthesis in two-stage fermentation or other metabolic applications.

The use of inducible promoters allows for the regulation of the expression of relevant genes, separating the cell growth process from the product production process. However, the potential toxicity, high cost, off-target effects, and delays in the translocation process of the inducers can greatly limit their application [4, 5]. A design using a biosensor induced by a specific metabolite can dynamically regulate the expression of related genes, but its versatility is limited [6]. In recent years, the quorum sensing system in synthetic biology has become increasingly valuable and can realize the dynamic expression regulation of related genes by sensing changes in cell density without the addition of exogenous inducers. That is, the bacteria will continuously synthesize and secrete autoinducer signaling molecules during the growth phase so that their abundance will increase with the cell concentration, and after reaching a certain concentration threshold, they will be able to respond in a real-time and precise manner [7, 8].

At present, the LuxI/R system of *Vibrio fischeri* has become the most representative quorum sensing mechanism in gram-negative bacteria [9]. One of these operons, luxICDABE, encodes the *luxI* synthase gene and the *luxCDABEG* luminescent gene and can be used for bioluminescence. Another operator, luxR, produces the quorum sensing regulatory protein LuxR, which is associated with the recognition of acylated homoserine lactone (AHL) signalers [10]. At low cell densities, luxI synthase maintains only background levels of expression and cannot activate the relevant gene circuits. When the N-(3-oxohexanoyl)-homoserine lactone (3OC6HSL) produced by luxI synthase accumulates to a certain threshold with increasing cell density, it induces fluorescence in the bacterial population [11, 12]. The quorum sensing system of *Pseudomonas aeruginosa* exhibits a sequential regulatory pattern [13], mainly including the Las and Rhl systems based on AHLs, as well as the quinolone system based on 2-alkyl-4 (1H) quinolone [2-alkyl-4 (1H) quinolone, AHQ] signaling molecules, which are independent of each other and can work synergistically [14, 15]. In addition, the mechanism of the *Agrobacterium tumefaciens* TraI/TraR system, which includes the traR, traAFB, traM, traCDG and traI-trb operons, has been gradually clarified [16]. When the cellular concentration reaches a certain threshold, AHLs produced by TraI synthase bind to TraR receptor proteins, and the formation of TraR-HSL activates the expression of traAFB, traCDG, and trb operons, which induces the replication of Ti plasmids and increases the chance of infection in the population [16–18].

With the clarification of the basic principle and core components of the QS system, it has been widely introduced into the study of metabolic regulation in synthetic biology. Our group constructed QS switches and an AND gate based on quorum sensing and applied the switches in the production of PHB and 5-aminolevulinic acid, effectively increasing the yield without causing cell damage [19, 20]. Soma et al. constructed QS-MTS by combining an adjustable density sensor based on the Lux system with a metabolic toggle switch, which transferred the metabolic flow from the central metabolic pathway to the isopropanol synthesis pathway and increased the isopropanol titer and yield by threefold and twofold, respectively [21]. Although gene circuits designed using the QS system can alleviate the problems of traditional regulatory strategies, they are mainly used for the regulation of the expression of single genes at a specific time, and problems involving the order of expression of the target genes and time intervals have not been effectively solved.

In cascade regulation in nature different genes are sequentially and continuously expressed in a certain order [22, 23], which provides a new idea for multigene temporal regulation in metabolic pathways. In prokaryotes, there exists a dual regulatory protein cascade with a relatively simple response mechanism. When an exogenous inducer is added, upstream regulatory proteins bind to the inducer and activate the expression of downstream regulatory proteins, which in turn activate the response of the target gene, ultimately realizing a sequential dynamic cascade of regulation. This cascade mechanism also has an activating effect that amplifies the response when regulating different cellular functions [24].

During metabolic pathway optimization, the maneuverability and precision of dual regulatory protein cascade circuits make them a fundamental model for the construction of novel gene regulatory circuits. For example, the *catabolic* gene of plasmid NAH7 in *Pseudomonas putida* is regulated by a NahR-XylS2 cascade [24]. In this circuit, the promoters are orthogonal. However, there is crosstalk between the signals, i.e., they can be activated by the same inducer salicylic acid. When there is no salicylic acid in the cell, the second regulatory protein XylS2 will only maintain a low background expression. When salicylic acid is added exogenously, the expression of the first regulatory protein NahR and the second regulatory protein XylS2 are activated sequentially, and the combination of the second regulatory protein XylS2 with salicylic acid can potentiate the activation of the P_m_ to achieve amplified expression of the target gene [24–26].

In this study, we constructed a self-induced dynamic temporal regulatory cascade circuit in *E. coli* using the QS system and dual regulatory protein cascade circuits, thus attempting to achieve precise expression of target genes in metabolic pathways at different times and nodes. Furthermore, a time interval library was constructed based on this circuit, which was verified at the protein level and transcription level, and the construction rules of the library were explored. Finally, the application value of the self-induced dynamic temporal regulation cascade circuit and the library was verified by the PHB production platform, which proved that the library can provide an adapted gene circuit to trigger expression of the target gene at different times in different orders.

## Results

### Construction of temporally regulated cascade circuits for exogenously induced expression

QS systems can optimize metabolic pathways in synthetic biology research by regulating cell growth and the response of associated protein levels [27, 28]. Due to the specificity of QS signaling molecules during signal transduction, individual bacteria accurately and rapidly recognize the corresponding signaling molecules in numerous microbial populations. However, many QS systems produce varying degrees of crosstalk when present in the same environment. For the purpose of temporal regulation, we screened the Las, Tra system and the Lux, Tra system to construct two cascade circuits. The Las and Tra systems can be activated by the same signaling molecule, and the complexes of the receptor proteins LasR and TraR upon binding to the signaling molecule will each specifically activate the corresponding promoter, i.e., the promoters are orthogonal. The same promoter orthogonal and signal crosstalk properties are also present in the Lux and Tra systems [29–31].

First, we verified the signal crosstalk effects of the Tra and Las systems and the Tra and Lux systems. The Las and Tra systems were found to have different degrees of response to N-(3-oxooctanoyl)-homoserine lactone (3OC8HSL) and N-(3-oxododecanoyl)-homoserine lactone (3OC12HSL), with the Las system having a higher degree of response induced by different concentrations of 3OC8HSL and 3OC12HSL and the Tra system having a higher degree of response induced by different concentrations of 3OC8HSL (Additional file 1: Figure S1a). Both the Lux and Tra systems also responded to 3OC6HSL, 3OC8HSL, and 3OC12HSL to varying degrees, with the Lux system and Tra system both responding to a lower degree of induction at different concentrations of 3OC12HSL (Additional file 1: Figure S1b). In general, the Las and Tra systems, as well as Lux and Tra, both exhibit signal crosstalk. Scott et al. studied the promoter crosstalk among Lux, Tra, Las and Rpa [29]. The regulatory proteins and promoters of these four QS systems were constructed on plasmids, combined in pairs, and induced with four HSL at different concentrations. The Las system and Tra system (Additional file 1: Figure S2a) and the Lux system and Tra system (Additional file 1: Figure S2b) all have orthogonal promoters.

**Fig. 1:**
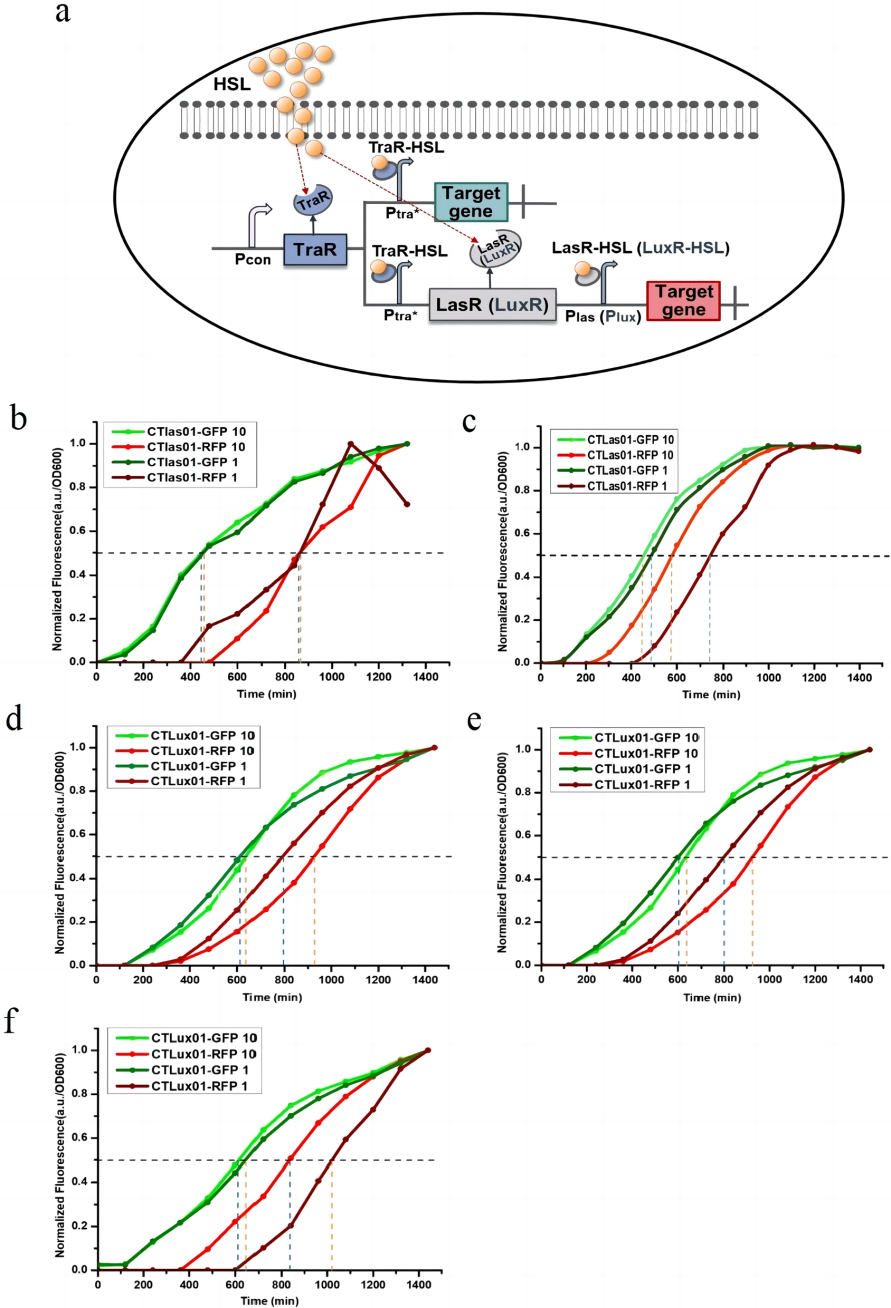
Exogenous addition of HSL induces the expression of temporally regulated cascade circuits. **a** Pattern diagram of the cascade circuits of exogenously induced expression. The hybrid promoter P_tra*_ was created by replacing the lux-box in the commonly used P_luxI_ promoter with the tra-box, which improves the inducibility of the Tra system [29]. **b** Normalized characterization curves for 3OC8HSL-induced Las, Tra system circuits. **c** Normalized characterization curves for 3OC12HSL-induced Las, Tra system circuits. **d** Normalized characterization curves for 3OC6HSL-induced Lux, Tra system circuits. **e** Normalized characterization curves for 3OC8HSL-induced Lux, Tra system circuits. **f** Normalized characterization curves for 3OC12HSL-induced Lux, Tra system circuits. To study the time interval between GFP fluorescent protein and RFP fluorescent protein expression in the regulatory circuit more intuitively and clearly, the fluorescence intensity of GFP and RFP after characterization was normalized, that is, the maximum value of each characterization curve was normalized. When the vertical coordinate of the characterization curve is 0.5, the value corresponding to the curve on the x-axis is the response time of the fluorescent protein [32, 33]

Therefore, we chose to use two sets of QS systems with orthogonal promoters and signaling crosstalk, Las, Tra and Lux, Tra, as the basis for the construction of cascade circuit strains CTLas01 and CTLux01, respectively, which require exogenous addition of HSL to induce expression. The regulation model of this circuit is shown in Fig. 1a, which is presented as an example of the Las, Tra system. Exogenous addition of 3OC8HSL or 3OC12HSL signaling molecules that bind to the receptor protein TraR to form the complex TraR-HSL activates P_tra*_, which in turn activates the expression of the first target gene, *gfp*. At the same time, P_tra*_ will activate a second receptor protein, LasR, which recognizes the same signaling molecules as TraR, and the complex LasR-HSL formed by the binding of LasR to the exogenous signaling molecules will potentiate the activation of P_las_, which in turn will activate the expression of the second target gene, *rfp*. Since there is a time interval between the activation of the two promoters, this allows the temporal regulation of the two target genes by exogenous addition of inducers. Then, the temporal expression function of the cascade circuit was verified, and the appropriate synthase gene was selected for the construction of the self-induced regulatory circuit. The results showed that the standardized response time interval of CTLas01 was 400 min with the addition of both 1 µM and 10 µM 3OC8HSL (Fig. 1b), while the standardized response time interval of CTLas01 was 250 min and 120 min with the addition of 1 µM 3OC12HSL and 10 µM 3OC12HSL, respectively (Fig. 1c). The cascade circuit constructed based on the Las, Tra system for exogenous addition of HSL-induced expression was shown to enable the temporal expression of genes. Moreover, we found that different concentrations of 3OC12HSL for induction could produce different time intervals, so *LasI*, which synthesizes 3OC12HSL, was selected as the HSL synthase gene for the construction of the self-induced dynamic temporal regulatory cascade circuit. The standardized response time intervals for the CTLux01 strain were approximately 160 min, 280 min, 200 min, 280 min, 380 min, and 220 min when 1 µM 3OC6HSL, 10 µM 3OC6HSL, 1 µM 3OC8HSL, 10 µM 3OC8HSL, 1 µM 3OC12HSL, and 10 µM 3OC12HSL were added, respectively (Fig. 1d, e, f). The cascade circuit constructed based on the Lux, Tra system for exogenous addition of HSL-induced expression was shown to achieve the temporal expression of gene. Different time intervals were obtained under the induction of different concentrations of 3OC6HSL, 3OC8HSL and 3OC12HSL, and we directly chose LuxI, which synthesizes 3OC6HSL, was selected as the HSL synthase gene for the construction of the self-induced dynamic temporal regulation cascade circuit.

### Construction of self-induced dynamic temporal regulation cascade circuits

**Fig. 2:**
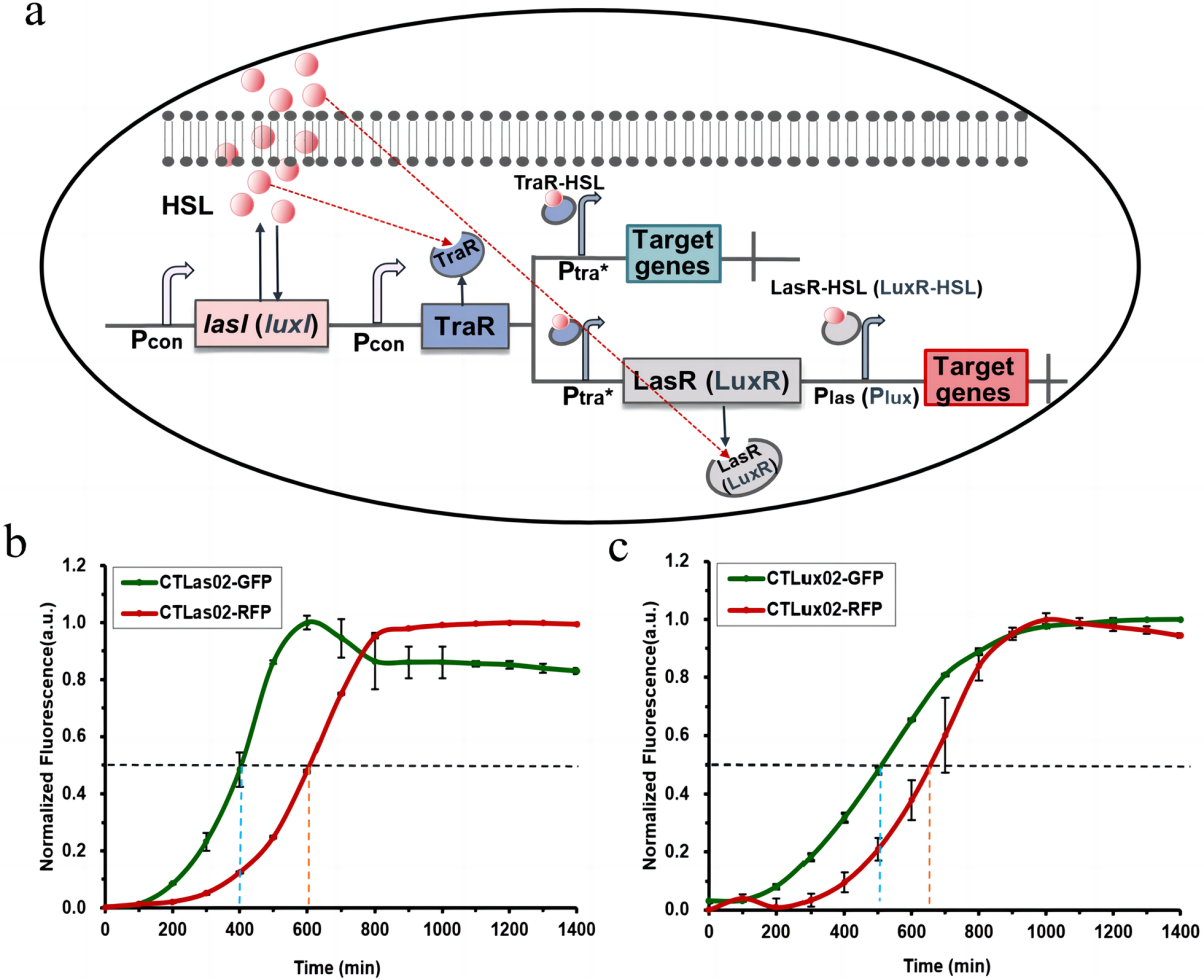
Construction of self-induced dynamic temporal regulation cascade circuits. **a** Pattern diagram of the self-induced dynamic temporal regulation cascade circuit. **b** Normalized characterization curves of self-induced circuits based on the Las, Tra system. **c** Normalized characterization curves of self-induced circuits based on the Lux, Tra system

We constructed two self-induced dynamic temporal regulation cascade circuit strains, CTLas02 and CTLux02, using *lasI*, which synthesizes 3OC12HSL, and *luxI*, which synthesizes 3OC6HSL, as autoinducer synthase genes, respectively. The specific regulatory mechanism is shown in Fig. 2a, which is presented as an example of the Las, Tra system. During bacterial growth, LasI synthase continuously synthesizes and secretes HSL signalers. When the cellular concentration reaches a certain threshold, HSL binds to the first receptor protein, TraR, and the resulting complex, TraR-HSL, can activate P_tra*_, which in turn activates the expression of the first target gene, *gfp*. Simultaneously, P_tra*_ activates the expression of the receptor protein LasR, which responds to the same HSL signaling molecule as the TraR receptor protein. Thus, the complex formed by the binding of LasR to HSL can potentiate the activation of P_las_, which in turn transcriptionally activates the expression of the second target gene, *rfp*. Since there is a time interval between the activation of the two promoters in the circuit, the expression of upstream and downstream genes in response to different time periods can be effectively achieved.

Characterization of the two strains of the self-induced dynamic temporal regulatory cascade circuit revealed a standardized response interval of 200 min for CTLas02 (Fig. 2b); CTLux02 had a standardized response interval of 150 min (Fig. 2c). This suggests that both self-induced dynamic temporal regulatory cascade circuits can realize the dynamic expression of upstream and downstream genes at different time intervals. Moreover, the expression intensity of both *gfp* and *rfp* in CTLas02 was found to be stronger than that in CTlux02, and the expression intensity of *rfp* in CTLux02 was very weak (Additional file 1: Figure S3). Since the expression intensity of the target gene has a greater impact on the circuit activation response, cascade circuits of the Las and Tra systems will be preferred for metabolic pathway modification at a later stage.

### Construction of a dynamic temporal regulation cascade circuit library

**Fig. 3:**
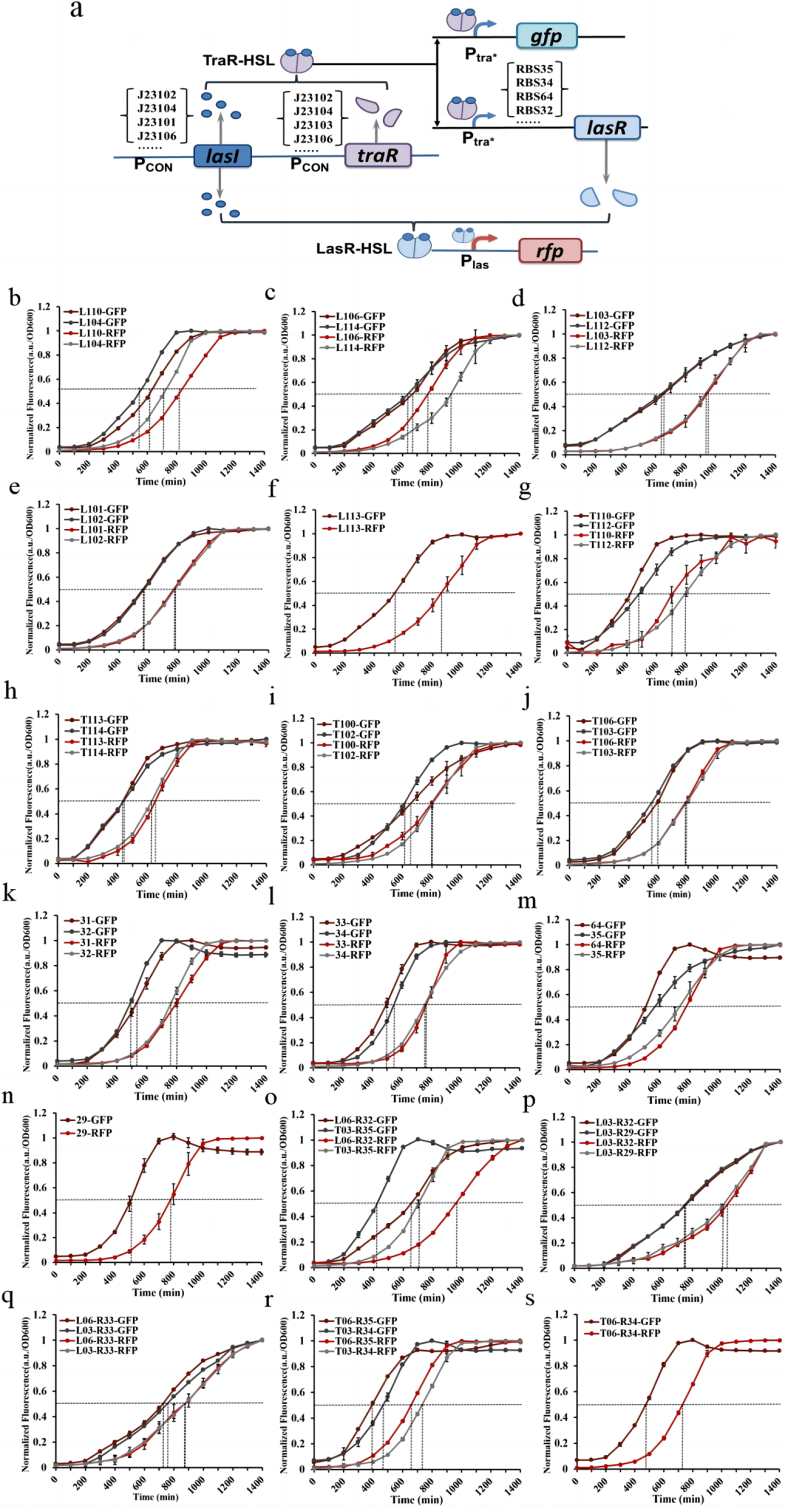
Construction of a dynamic temporal regulation cascade circuit library. **(a)** Pattern diagram of the dynamic temporal regulation cascade circuit library construction strategies. **b-f** Normalization curves of cascade circuits regulating the expression intensity of LasI synthase. **g-j** Normalization curves of cascade circuits regulating the expression intensity of TraR receptor protein. **k-n** Normalization curves of cascade circuits regulating the expression intensity of LasR receptor protein. **o-s** Normalized curves of cascade circuits for combined regulation of expression intensity

To provide more alternative generic components for metabolic pathway modification in synthetic biology, we utilized promoter engineering and RBS replacement for the construction of cascade circuit libraries for dynamic temporal regulation. A series of dynamically regulated circuits with different time intervals were obtained by regulating the expression intensity of the three core components of the circuits, LasI synthase, TraR receptor protein and LasR receptor protein (Fig. 3a).

First, we performed independent regulation of the expression strength of each element. IGEM provides promoter (Additional file 2: Table S1) and RBS sequences (Additional file 3: Table. S2), and we chose different strengths of promoters and RBSs for replacement separately. The expression intensity of LasI synthase, the first core regulatory element of CTLas02, is regulated by promoters J23100 and RBS34. We selected high-strength (J23102, J23104, J23101), medium-strength (J23106, J23110), and low-strength (J23113, J23103, J23112, J23114) promoters for the replacement of J23100 and found that strains L110, L104, L106, L114, L103, L112, L101, L102, and L113 had normalized response time intervals of 215 min, 160 min, 110 min, 310 min, 290 min, 300 min, 210 min, 205 min, and 305 min, respectively (Fig. 3b-f). Compared with CTLas02 without circuit modification (time interval of 200 min), the normalized response time interval of LasI synthase after promoter replacement was expanded both before and after 200 min, indicating that the expression intensity of LasI synthase can be efficiently regulated by promoter engineering. The expression intensity of TraR receptor protein, the second core regulatory element of CTLas02, is regulated by promoters J23104 and RBS30. We selected high-strength (J23100, J23102), medium-strength (J23106, J23110), and low-strength (J23114, J23113, J23103, J23112) promoters for the replacement of J23104 and found that strains T100, T102, T113, T114, T110, T112, T106, and T103 had normalized response time intervals of 145 min, 200 min, 210 min, 185 min, 280 min, 310 min, 185 min, and 240 min, respectively (Fig. 3g-j). The expression intensity of LasR receptor protein, the third core regulatory element of CTLas02, is regulated by promoters P_tra*_ and RBS30. Therefore, we selected a series of RBSs (RBS31, RBS32, RBS33, RBS34, RBS64, RBS35, RBS29) with different expression intensities to replace RBS30. The normalized response time intervals for strains 31, 32, 33, 34, 64, 35 and 29 were found to be 280 min, 265 min, 285 min, 210 min, 260 min, 165 min and 270 min, respectively (Fig. 3k-n). Finally, we replace the promoter and RBS combinations to construct a cascade circuit library with a wider range of time intervals. After characterization, we found that the normalized response time intervals for strains L06-R32, T03-R35, L03-R32, L03-R29, L06-R33, L03-R33, T06-R35, T03-R34 and T06-R34 were 310 min, 275 min, 290 min, 270 min, 140 min, 120 min, 260 min, 267 min and 255 min, respectively (Fig. 3o-s). The combinatorial construction did not effectively extend the range of time intervals but only increased the diversity of time intervals of the cascade circuits. We obtained a library of dynamic temporal regulation cascade circuits with time intervals ranging from 110 to 310 min, which contains 34 different circuit modifications.

**Fig. 4:**
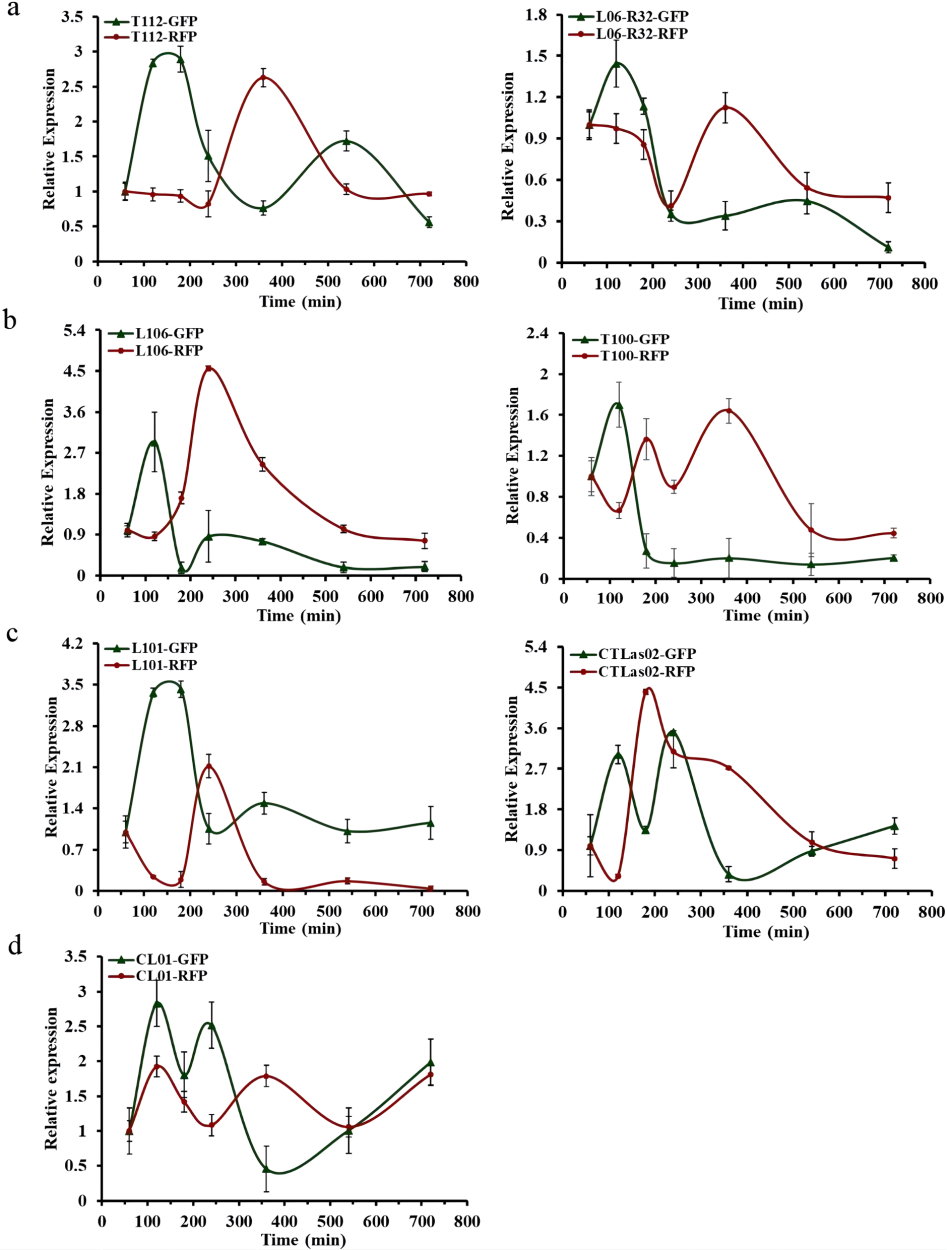
Validation of cascade circuit libraries at the transcriptional level. **a** Large time interval circuit. **b** Small time interval circuit. **c** Intermediate time interval circuit. **d** Circuit with no time interval. The strains were continuously cultured in a constant temperature microplate shaker for 12 h. Samples were taken at 1 h, 2 h, 3 h, 4 h, 6 h, 9 h, and 12 h and then subjected to real-time fluorescence quantitative PCR

Next, we selected representative cascade circuits with larger, smaller and intermediate time intervals in the library for transcriptional level studies. The larger (T112, L06-R32), smaller (L106, T100) and intermediate time interval (L101, CTLas02) strains were used as experimental groups, and strain CL01 without a time interval was used as a control group. The response time intervals at the transcriptional level were 170 min and 180 min for T112 and L06-R32 (Fig. 4a), 50 min and 60 min for L106 and T100 (Fig. 4b), and 105 min and 50 min for L101 and CTLas02 (Fig. 4c), respectively. The control group CL01 was present at the transcriptional level without circuit response time interval (Fig. 4d). Although the time intervals at the protein level are not exactly the same as those at the transcription level, the magnitude of the time interval trends presented by them is the same, suggesting that the constructed cascade circuit libraries can provide a series of gene circuits with different time intervals and different expression intensities. Among them, the time interval of CTLas02 was 50 min, which was not in line with the expected results, probably because the sampling interval of the first 3 h was large and could not accurately reveal the expression of fluorescent protein genes in the circuits.

To facilitate the expansion of the library in metabolic regulation at a later stage, we studied the relationship between the time intervals of different circuits and the promoter and RBS strength of the core element. The promoter and RBS intensities were compared to various time differences in the library, and a linear regression trendline was fitted to analyze the experimental results. When studying the regulation of LasI synthase expression strength, one data point was excluded, which had a large degree of dispersion. Then, an extremely strong negative correlation was found between promoter strength and time interval (Additional file 1: Figure S4a). When studying the regulation of TraR receptor protein expression strength, a weak negative correlation between promoter strength and time interval was found (Additional file 1: Figure S4b). Finally, studying the regulation of LasR receptor protein expression intensity revealed that there was also an extremely strong negative correlation between RBS strength and time interval (Additional file 1: Figure S4c).

### Application of a library of dynamic temporal regulation cascade circuits

**Fig. 5:**
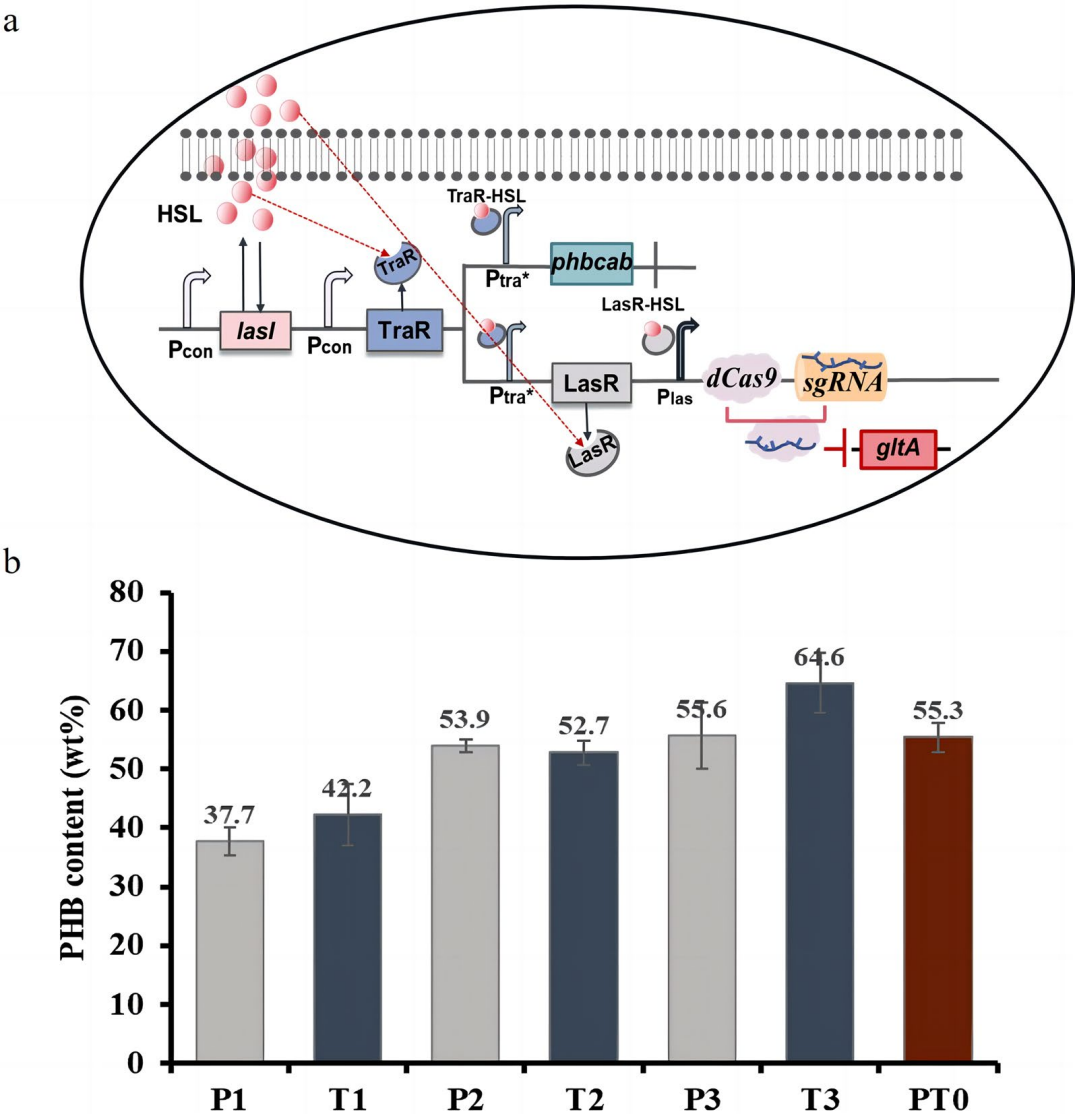
Application of a self-induced dynamic temporal regulation cascade circuit. **a** Diagram of the CRISPRi system combined with a self-induced dynamic temporal regulation cascade circuit for PHB production (PHB production followed by inhibition of the TCA cycle is introduced as an example. During the growth of *E. coli*, 3OC12HSL secreted by LasI synthase binds to TraR after reaching a certain concentration threshold, and the complex formed activates P_tra*_, which in turn activates *phbcab*. At the same time, P_tra*_ also activates LasR, and the complex formed upon binding to 3OC12HSL potentiates the activation of P_las_, which then activates the transcription of *dCas9* and *sgRNA*. The bound complex targets and represses the expression of *gltA* in the genome, which in turn represses the TCA cycle and promotes the flux of more acetyl-CoA into the PHB production pathway). **b** PHB content of fermentation strains

PHB is a valuable biomaterial that is biocompatible and biodegradable [34, 35]. During PHB production, the precursor substance acetyl-CoA both maintains normal product synthesis and some enters the tricarboxylic acid cycle (TCA cycle).The TCA cycle is one of the most critical processes in central metabolism, so it is impossible to directly knock out this pathway to improve the production capacity of PHB, which will lead to a conflict between bacterial growth and PHB synthesis [36, 37]. Therefore, we chose the unmodified time interval circuit in the library for application to PHB production, i.e., we introduced the CRISPRi system on the basis of the temporal regulatory cascade circuit to realize the redirection of metabolic flow, which separated the growth phase of the strain from the production phase so that more of the precursor would flow to the production pathway in the appropriate phase (Fig. 5a).

When constructing control strains, it was necessary to keep the expression intensity of the production genes consistent between the control and experimental groups, so we screened for constitutive promoters with similar intensities to the QS promoters P_tra*_ and P_las_. We screened a series of constitutive promoters to construct characterization plasmids and selected J23111 and J23102 as control promoters for P_las_ and P_tra*_, respectively, to construct blank control fermentation strains (Additional file 1: Figure S5). We chose *gltA* to achieve effective inhibition of the TCA cycle and *phbcab* for PHB production and constructed three groups of fermentation strains according to the different expression orders of these two target genes. The first group, which was subjected to PHB production followed by inhibition of TCA cycle, consisted of a blank control group P1 without cascade circuits (J23102 controlling *phbcab* expression), a negative control group P2 containing only cascade circuits (P_tra*_ controlling *phbcab* expression), and an experimental group P3 combining cascade circuits with the CRISPRi system (P_tra*_ controlling *phbcab* expression and P_las_ controlling *gltA* expression) (Additional file 1: Figure S6a). The second group, which was subjected to inhibition of TCA cycling followed by PHB production, consisted of a blank control T1 without cascade circuits (J23111 controlling *phbcab* expression), a negative control T2 containing only cascade circuits (P_las_ controlling *phbcab* expression), and an experimental group T3 in which cascade circuits were combined with the CRISPRi system (P_tra*_ controlling *gltA* expression and P_las_ controlling *phbcab* expression)(Additional file 1: Figure S6b). The third group is the experimental group PT0 with both TCA cycle inhibition and PHB production (P_tra*_ controls both *phbcab* and *gltA* expression) (Additional file 1: Figure S6c).

The growth status of the experimental group and the control group was maintained in a good state. In the first group, P2 had the best growth status, while in the second group, T2 had the best growth status (Additional file 1: Figure S7). P2 and T2 also had the highest glucose consumption (Additional file 1: Figure S8). In the first group, the PHB content of P3 was 55.6%, which was 1.5 times that of the blank control group P1 and 1 times that of the negative control group the P2. In the second group, the PHB content of T3 was 64.6%, which was 1.5 times that of the blank control group T1 and 1.2 times that of the negative control group T2. The PHB content of PT0 was less than that of P3 and T3 (Fig. 5b). This indicates that the production strains constructed based on a dynamic temporal regulation cascade circuit with the CRISPRi platform can effectively increase the PHB content. In addition, the results of real-time fluorescence quantitative PCR revealed that the downregulation of *gltA* in the experimental groups P3 and T3 started at 9 h and 3 h, respectively, whereas the downregulation of *gltA* in the control P2 and T2 started at 12 h and 9 h, respectively (Additional file 1: Figure S9). It is further illustrated that the CRISPRi system can target and repress the expression of *gltA* in the genome at specific time intervals in this circuit, thereby increasing the content of PHB.

**Fig. 6:**
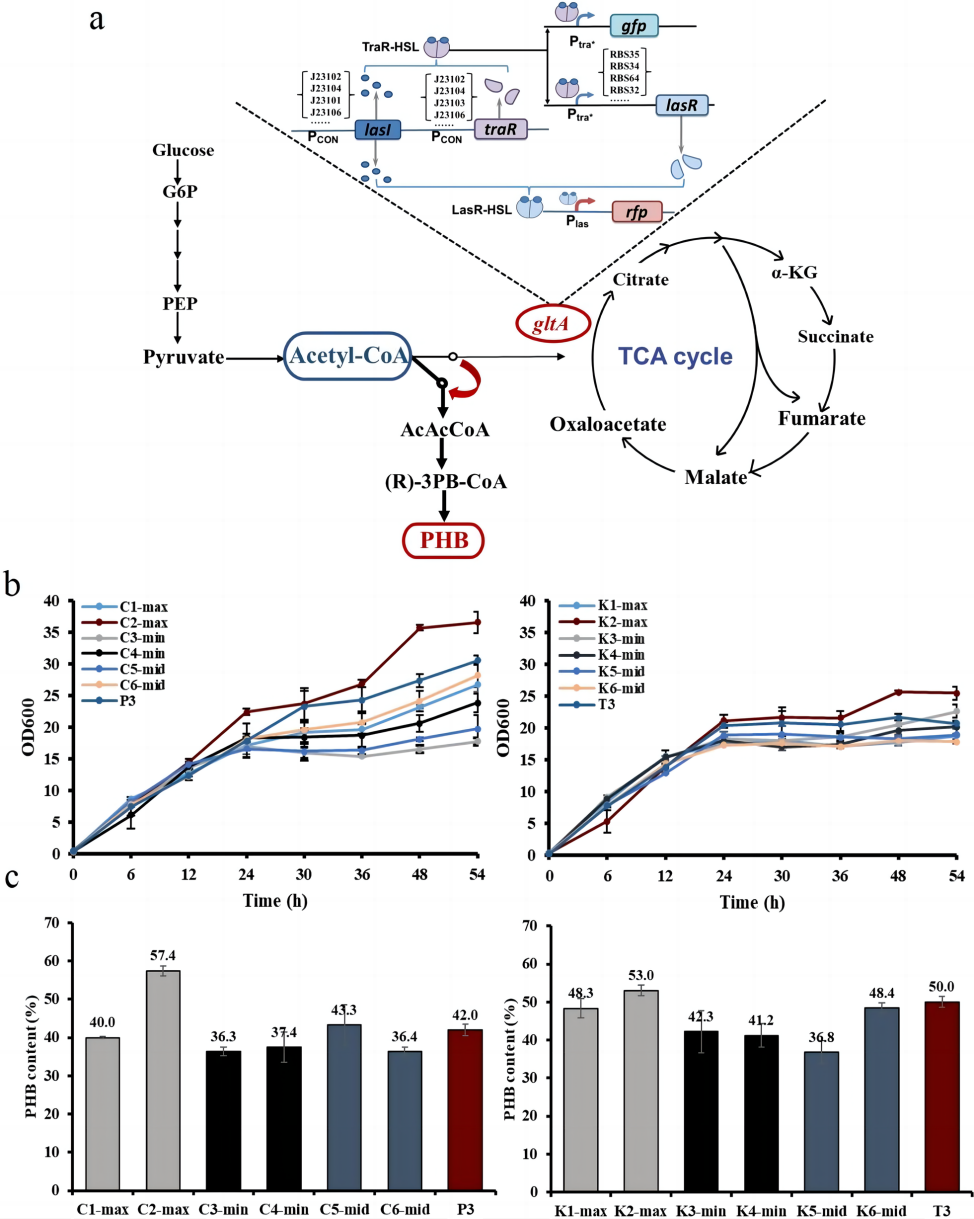
Application of the dynamic temporal control cascade circuit library. **a** The metabolic pathway to the synthetic product. **b** Growth of fermented strains. **c** PHB content of fermentation strains

We have demonstrated the effectiveness of the unmodified cascade circuits in the library in PHB production, and next, we will further select a small number of representative time-interval circuits in the library to explore the effect of upstream and downstream gene expression at different time intervals on PHB synthesis and then select adapted gene circuits to optimize the production (Fig. 6a).

When cascade circuits are used in chemical production, the time interval and the expression intensity of the target gene have a crucial impact on production. To more precisely study the effects of different time intervals on chemical production efficiency, we screened a series of representative time interval circuits based on keeping the expression intensity in a certain range, including L112 (300 min) and L06-R32 (310 min) with large time intervals, L104 (160 min) and L106 (110 min) with small time intervals, and 34 (210 min) and T06-R34 (260 min) with intermediate time intervals (Additional file 1: Figure S10). The fermentation strains P3 and T3 were used as templates, and the corresponding fermentation strains were constructed according to the construction of the screened larger, smaller and intermediate time interval circuits, i.e., the promoters and RBSs of the three core elements in the strains were replaced according to the correspondences of the strains with different time interval circuits. The two groups were divided according to the difference in the order of expression of *gltA* and *phbcab*. The first group, which was subjected to PHB production followed by TCA cycling inhibition, consisted of strains with P_tra*_ controlling the expression of *phbcab* and P_las_ controlling the repression of *gltA*, including C1-max, C2-max, C3-min, C4-min, C5-mid, and C6-mid (hereafter referred to as group C). The second group, in which the TCA cycle was inhibited before PHB production, consisted of strains with P_tra*_ controlling the repression of *gltA* and P_las_ controlling the expression of *phbcab*, including K1-max, K2-max, K3-min, K4-min, K5-mid, and K6-mid (hereinafter referred to as group K).

After PHB fermentation, the growth trends of all strains were very similar, but the overall growth trend of group C was better than that of group K (Fig. 6b). Among them, the C2-max strain showed the best growth, and the glucose consumption of C2-max was also the highest among all the strains (Additional file 1: Figure S11). The highest PHB content of C2-max reached 57.4% in group C. The highest PHB content of K2-max reached 53% in group K (Fig. 6c). We found that circuits with different time intervals had different PHB production effectiveness, and that the superior production circuit strain C2-max was obtained by screening.

## Discussion

Metabolic engineering enables the efficient synthesis of biobased products by modifying the metabolic pathways of microbial cell factories and has been widely used in the pharmaceutical, environmental and fermentation industries [38, 39]. Emerging dynamic regulatory strategies can effectively address the problems of the accumulation of toxic intermediate metabolites and of growth retardation through precise regulation [40–42]. To further expand the application scope of dynamic regulation strategy and realize precise regulation at different times and in different sequences, we have for the first time combined dual- regulatory protein cascade circuits with a QS module, combined with promoter engineering and RBS replacement to construct a series of time interval circuits and explore their application potential.

Our constructed self-induced dynamic temporal regulatory circuit can realize the dynamic expression of target genes at different time periods. We then optimized and modified the circuit to provide more selectable generic components for synthetic biology for metabolic applications. In the optimization of metabolic engineering gene circuits, precise and efficient dynamic regulation strategies play a crucial role in determining the research progress [43, 44]. Different promoters can determine the regulation pattern and expression intensity of genes, which are among the crucial factors affecting gene expression levels. There are two common approaches to promoter engineering modification: (1) combining promoters with transcription factors or mutating endogenous promoters of target genes [45] or (2) directly replacing promoters in dynamically regulated gene circuits [20]. Liu et al. replaced the promoter P_ede BGC_ with P_mwp_, P_spc_, P_xylA_, P_shuttle-09_, P_grac_, and P_43_ and significantly increased the production of imidacloprid in *Brevibacillus brevis* X23 [46]. In addition, promoter engineering is usually combined with RBS optimization to modify the gene expression regulation mode. Sarah et al. constructed a gene toolbox of optimized circuits for the regulation of key genes in *Bacillus subtilis* using a random mutation library of three superior RBS sequences and an endogenous constitutive promoter [47]. Therefore, we also utilized promoter engineering and RBS replacement to regulate the expression intensity of the three core components in the temporal control circuit and constructed a time interval circuit library. During the construction of the tool library, we first performed intensity modulation of individual core elements (Fig. 3 b-n). After obtaining a series of circuits with different time intervals, an attempt to extend the range of time intervals by regulating the promoter and RBS simultaneously was unsuccessful (Fig. 3 o-s). Because the time interval is determined by the expression times of the two target genes, which are correlated with the expression intensities of LasI, TraR, and LasR, the pattern of circuit regulation when the intensities of the two elements are simultaneously varied is more complex than that of a single element, and needs to be screened for a wider range of time differences in a sufficiently large number of combinations. However, in the process of plasmid construction, when some promoters and RBS were replaced at the same time, monoclones could not be successfully screened, resulting in the number of plasmids being limited and a wider range of time intervals could not be obtained. When correlation studies between the intensity of LasI synthase expression and time interval were performed, a moderate correlation between promoter intensity and time interval was found (Pearson = 0.55). However, there is a point in the fitted curve with a large degree of dispersion, promoter J23106. Because of our limited sample size and the large dispersion of this value, this point also has a large impact on the curve correlation analysis. It is speculated that when promoters of different strength are applied to the circuit, they have different activation effects on the target genes in the circuit, and thus cause this result. However, the other promoters showed a trend that the higher the intensity, the smaller the time interval. Therefore, we consider that the presence of this point affects the correlation analysis between the two variables. In order to better fit the linear relationship, we chose to remove this point with a large degree of dispersion and performed a relatively accurate correlation analysis, which revealed an extremely strong negative correlation between them (Pearson = 0.9).

We chose a valuable biomaterial, PHB, to initially validate the circuit. During the production of PHB, a large portion of the precursor substance acetyl-CoA is diverted to the TCA cycle, reducing PHB production. Therefore, we attempted to utilize temporal regulatory circuits to activate *phbcab* expression and inhibit TCA cycling so that precursors flow more into the PHB production pathway at the appropriate stage. Currently, the emerging CRISPRi system provides some basis for gene transcription regulation [48]. This system requires only the design of a specific guide RNA (sgRNA) for the target gene to accomplish simple target regulation [49], and it has been widely used in transcriptional regulation in various bacteria, fungi and mammals [50, 51]. Therefore, we combined the CRISPRi system with a dynamic temporal regulation cascade circuit to ameliorate the carbon shunting problem and achieve increased yield. After fermentation, we found that the P3 PHB content was the highest in the first group, the T3 PHB content was the highest in the second group, and the PHB content of PT0 was slightly lower than that of T3 (Fig. 5 b). This indicates that the combination of dynamic temporal regulatory cascade circuits and the CRISPRi system can effectively separate the growth stage from the production stage of bacteria and increase the PHB content. Moreover, we found that the contents of P3 and T3 were different, indicating that the different expression orders of the two target genes, *phbcab* and *gltA*, would affect the PHB content. In addition, we found that P2 had the best growth trend and the highest sugar consumption in the first group, but the PHB content was not the highest, and the same result was observed in the second group. Theoretically, the better the cell's growth state, the greater the glucose consumption and thus the higher the PHB content. However, P1 and T1 are regulated by constitutive promoters that regulate the expression of production genes, and earlier expression increases the metabolic burden [20], which in turn affected growth and led to the lowest PHB levels. In contrast, P2 and T2 introduced a self-induced temporal regulatory circuit to start PHB synthesis at the appropriate stage and reduce the metabolic burden, so they had the best growth trend and maximum glucose consumption. The growth trend and glucose consumption of P3 and T3 were lower than those of P2 and T2. This is because P3 and T3 introduced the CRISPRi system along with the self-induced temporal regulatory circuit, and the CRISPRi system creates a certain amount of metabolic stress, which affects the growth of the strains and consequently decreases glucose consumption. However, P2 and T2 had lower PHB contents than P3 and T3. This is because the CRISPRi system introduced by P3 and T3 effectively suppressed the expression of *gltA* in the genome and increased the content of PHB. In addition, the minimal glucose consumption of P3 and T3 also suggests that the combination of the temporal regulatory circuit with the CRISPRi system not increases PHB content but also provides good cost savings.

In dynamic regulation studies, the model of metabolic flux regulation often selects related chemicals with acetyl-CoA as the precursor, but most of the studies use “on–off” dynamic regulatory element [52, 53]. This element requires appropriate induction timing, and switching to production mode early or late may affect bacterial growth and target chemical synthesis. Therefore, we believe that the study of the interval time and the order of regulation in the dynamic regulation is of practical application to improve the efficient synthesis of the products. We reapplied the library to PHB production and found that different time interval circuits have different potentials in PHB production. We performed a screen and identified the better circuit-producing strain C2-max, which showed a significant increase in PHB content compared with the unmodified P3 strain, which can reach 57.4%. Combined with the systematic errors in PHB fermentation experiments, these results confirmed that libraries can be used to screen for adapted circuits with better time intervals to be subjected to metabolic pathway modification to further improve the productivity of PHB.

## Conclusion

Dynamic regulation is a commonly used strategy for the efficient cell synthesis of biobased chemicals. However, the strategy of dynamically regulating multiple genes at different times and in different orders is still not effectively solved. We used a quorum sensing system and a dual-regulatory protein cascade circuit to construct a self-induced dynamic temporal regulation cascade circuit, and based on this circuit, we generated a cascade circuit library. Using the PHB production platform, combined with CRISPRi technology, the application value of the circuit and the library was verified, which improved the time interval circuit for PHB production. The construction and application rules of the time interval library were also explored, which provided new research ideas and tools for the accurate allocation of metabolic flux.

## Materials and methods

### Strains and culture medium

All Strains used in this study are listed (Additional file 4: Table S3). Luria − Bertani (LB) broth (5 g/L yeast extract, 10 g/L tryptone, 10 g/L NaCl) was used for plasmid cloning; LB agar (LB broth supplemented with 15 g/L agar powder) was used for plasmid construction and screening. To maintain plasmids and screen recombinants, antibiotics (chloramphenicol (25 μg/mL), kanamycin (50 μg/mL), ampicillin (100 μg/mL), and spectinomycin (50 μg/mL)) were used. 3OC6HSL (K3255; Sigma), 3OC8HSL (O1764; Sigma) and 3OC12HSL (O9139; Sigma) were added as inducers if needed. For PHB fermentation experiments, we used modified M9 medium (15.6 g/L Na_2_HPO_4_·12H_2_O, 3 g/L KH_2_PO_4_, 1 g/L NH_4_Cl, 1 g/L NaCl, 0.24 g/L MgSO_4_, 0.015 g/L CaCl_2_, 2 g/L yeast extract) supplemented with 50% glucose solution to 20 g/L as the carbon source.

### Plasmid construction

All plasmids (Additional file 5: Table S4) and primers (Additional file 6: Table S5) in this article are listed separately. Primers were synthesized for plasmid construction through PCR and Gibson assembly [54]. Detailed methods for plasmid construction are described in the Additional file.

## Quorum sensing system characterization

pBW400-LasR-P_las_-GFP, pBW213-TraR-P_tra*_-GFP, and pBW313-LuxR-P_lux_-GFP were transfected into *E. coli* TOP10 to obtain strains Las01, Tra01, and Lux01, respectively, which validated the signaling crosstalk of the QS system. Single colonies were transferred to 5 mL of LB broth with appropriate antibiotics. Following culture at 220 rpm/min and 37 °C for 12 h, 2% seeds were inoculated into a 24-well microassay plate containing 1 mL of LB medium supplemented with appropriate antibiotics. Then, 3OC6HSL, 3OC8HSL, and 3OC12HSL corresponding to different concentrations (0, 1 × 10^–3^, 1 × 10^–2^, 1 × 10^–1^, 1, and 10 µM) were added, and a negative control group was set up with the addition of only 1 mL of LB liquid medium. The 24-well plate was cultured at 37 °C under vigorous shaking. After dilution and washing, the cell density at OD_600_ and green fluorescence (excitation at 485 nm and emission at 528 nm) were detected after a 6 h inoculation using a Multi-Detection Microplate Reader (Synergy HT, Biotek, USA).

### Quorum sensing circuit characterization

The plasmids pBW213-TraR-P_tra*_-LasR-GFP and P_las_-RFP (Las, Tra) and pBW213-TraR-P_tra*_-LuxR-GFP and P_lux_-RFP (Lux, Tra) were co-transfected into *E. coli* TOP10 to obtain the exogenously added inducer-expressing QS circuit strains CTLas01 and CTLux01. Plasmids pBW213-LasI-TraR-P_tra*_-LasR-GFP and P_las_-RFP (Las, Tra) and pBW213-LuxI-TraR-P_tra*_-LuxR-GFP and P_lux_-RFP (Lux, Tra) were co-transfected into *E. coli* TOP10 to obtain the self-induced temporally regulated circuit strains CTLas02 and CTLux02. Single colonies were subsequently transferred to 5 mL of LB broth with appropriate antibiotics. Following culturing at 220 rpm/min and 37 °C for 12 h, 2% of the seeds were washed and inoculated into a 24-well plate, with a total volume of 1 mL of LB medium. Antibiotics were added, 1 mL of LB liquid medium was used as a blank control group, and the 24-well plates were incubated for 24 h with continuous vigorous shaking. The cell density at OD_600_, green fluorescence (excitation at 485 nm and emission at 528 nm) and red (excitation at 585 nm and emission at 640 nm) fluorescence were detected using a Multi-Detection Microplate Reader (Synergy HT, Biotek, U.S.). In addition, CTLas01 and CTLux01 characterization required exogenous addition of the corresponding 3OC6HSL, 3OC8HSL and 3OC12HSL.

### Construction of a dynamic temporal regulation cascade circuit library

We obtained a series of plasmids with different expression strengths by replacing different promoters and RBSs, which were co-transfected with P_las_-RFP into *E. coli* TOP10 to construct a time interval library. Moreover, plasmids J23110-RFP, J23113-RFP, J23103-RFP, J23112-RFP, 35-RFP, 34-RFP, 64-RFP, 32-RFP, 29-RFP, 31-RFP, and 33-RFP were obtained by the inverse PCR construction method using J23102-RFP as the template and were used for exploring the construction rule of the library.

### Quantitative real-time PCR (RT‒PCR)

Following the instructions of the FastQuantity RT Kit (KR106) (Tiangen Biochemical Technology Co., Ltd., Beijing, China), total RNA was extracted from *E. coli* cells at different growth stages. Then, the concentration of extracted RNA was determined by a Nanodrop 2000 (Thermo Scientific Inc., Wilmington, USA). cDNA was obtained following the instructions of the First Strand cDNA Synthesis Kit (TOYOBO). The PCR included 12.5 μL of SYBR Green Real-time PCR Master Mix (TOYOBO), 2.5 μL of diluted cDNA (500 ng/μL) reaction mixture, 1 μL each of forward and reverse primer (10 μmol) and 8.0 μL of ddH_2_O. The RT‒PCR assays were performed on a 7900HT Fast Real-Time PCR System (Applied Biosystems, Carlsbad, California, USA). The relative gene expression was calculated by the 2^(‑ΔΔCt)^ method [55].

### PHB fermentation

Fermentation-associated plasmids were constructed as described in the supplementary material to obtain P1, P2, P3, T1, T2, T3, and PT0 (Additional file 1: Figure S6). The promoter and RBS of the fermentation strain were replaced to obtain C1-max, C2-max, C3-min, C4-min, C5-mid, C6-mid, K1-max, K2-max, K3-min, K4-min, K5-mid and K6-mid. Individual strains were inoculated into LB liquid medium containing 5 mL of corresponding antibiotics and cultured overnight at 220 rpm/min at 37 °C. The seed solution was transferred to 50 mL of M9 liquid medium at a 5% inoculum volume, and the corresponding antibiotics, 2 mL of glucose, 100 μL of 1 M MgSO4, and 50 μL of 0.1 M CaCl_2_ were added. The flask was placed in a shaker at 37 °C and 220 rpm/min for 54 h, and an appropriate amount of fermentation liquid was taken every 6 h for the detection of OD_600_ and glucose content. When the glucose content was less than 10 g/L, supplementation was promptly performed. The OD_600_ was measured with an ultraviolet‒visible spectrophotometer, and 15 μL of fermentation culture supernatant was used to analyze glucose consumption in a biosensor analyzer. The PHB content was determined by gas chromatography (GC).

### Measurement of PHB production

After fermentation, the cells were harvested by centrifugation at 7000 rpm for 15 min. The bacteria sample was lyophilized for 6 h, and the cell sample was weighed. The bacteria were ground carefully, and 15 mg of the bacteria sample was accurately weighed into a well-sealed small bottle. The weight of the bottle with the bacteria sample was weighed. Concentrated sulfuric acid (150 μL), 850 μL of methanol and 1 mL of trichloromethane were added in that order under a fume hood. The bottles were sealed and placed into a 100 °C oil bath for 1 h. The bottles were then removed, and 1 mL of ultrapure water was added. The bottles were sealed, and the samples were thoroughly mixed by vortex oscillation. The samples were left standing for at least an hour until the liquid separated. The top water phase and lower organic phase were stored. PHB dissolved into the organic solvent. The organic phase (~ 600 μL) was carefully transferred to a 1 mL vial for a GC analysis to determine the content of PHB [56].

### Supplementary Information

**Additional file 1: Figure S1.** Verification of signal crosstalk in QS systems. (a) Characterization of signal crosstalk in Las and Tra systems. (b) Characterization of signal crosstalk in Lux and Tra systems. **Figure S2.** Verification of promoter orthogonality in QS systems. (a) Characterization of promoter orthologs in the Las, Tra system. (b) Characterization of promoter orthologs in the Lux, Tra system. **Figure S3**. Construction of self-induced dynamic temporal regulation cascade circuits. (a) Characterization of self-induced dynamic temporal regulation cascade circuits based on Las, Tra systems. (b) Characterization of self-induced dynamic temporal regulation cascade circuits based on Lux, Tra systems. **Figure S4.** The rule between the time interval of the circuit and the strength of the promoter, RBS. (a) The relationship between promoter strength and the time interval obtained by regulating LasI. (b) The relationship between promoter strength and the time interval obtained by regulating TraR. (c) The relationship between RBS strength and the time interval obtained by regulating LasR. **Figure S5.** Screening of constitutive promoters in PHB fermentation. **Figure S6.** Construction of dynamic temporal regulation cascade circuit fermentation strains. (a) PHB production followed by inhibition of TCA cycle. (b) Inhibition of TCA cycle followed by PHB production. (c) Inhibition of TCA cycle and production of PHB simultaneously. **Figure S7.** Growth of fermented strains. **Figure S8.** Glucose consumption of fermenting strains. **Figure S9.** The effect of CRISPRi system on *gltA*. **Figure S10.** Characterization of dynamic temporal control cascade circuit library. **Figure S11.** Glucose consumption of fermenting strains. (a) PHB production followed by inhibition of TCA cycle. (b) Inhibition of TCA cycle followed by PHB production.

**Additional file 2: Table S1.** Constitutive promoter strength and sequence.

**Additional file 3: Table S2.** RBS strength and sequence.

**Additional file 4: Table S3.** Strains used in this study.

**Additional file 5: Table S4.** Plasmids used in this study.

**Additional file 6: Table S5.** Primers used in this study.

## Data Availability

All data generated or analyzed during this study are included in this published article.

## Abbreviations

3OC6HSL: N-(3-oxohexanoyl)-homoserine lactone
3OC8HSL: N-(3-oxooctanoyl)-homoserine lactone
3OC12HSL: N-(3-oxododecanoyl)-homoserine lactone
AHL: Acyl-homoserine lactone
Amp: Ampcillin
Cm: Chloromycin
Kan: Kanamycin
Spc: Spectinomycin
GC: Gas chromatography
OD: Optical density
PHB: Poly-β-hydroxybutyric acid
QS: Quorum sensing
RBS: Ribosome Binding Site
TCA: Tricarboxylic Acid Cycle
