# Supplementary material for: Construction of cascade circuits for dynamic temporal regulation and its application to PHB production
Source: Biotechnol Biofuels Bioprod. 2023 Oct 27;16:158. doi: 10.1186/s13068-023-02416-x (PMC10604415; doi:10.1186/s13068-023-02416-x)
Supplement: Supplementary file 1 — Additional file 1: Figure S1. Verification of signal crosstalk in QS systems. (a) Characterization of signal crosstalk in Las and Tra systems. (b) Characterization of signal crosstalk in Lux and Tra systems. Figure S2. Verification of promoter orthogonality in QS systems. (a) Characterization of promoter orthologs in the Las, Tra system. (b) Characterization of promoter orthologs in the Lux, Tra system. Figure S3. Construction of self-induced dynamic temporal regulation cascade circuits. (a) Characterization of self-induced dynamic temporal regulation cascade circuits based on Las, Tra systems. (b) Characterization of self-induced dynamic temporal regulation cascade circuits based on Lux, Tra systems. Figure S4. The rule between the time interval of the circuit and the strength of the promoter, RBS. (a) The relationship between promoter strength and the time interval obtained by regulating LasI. (b) The relationship between promoter strength and the time interval obtained by regulating TraR. (c) The relationship between RBS strength and the time interval obtained by regulating LasR. Figure S5. Screening of constitutive promoters in PHB fermentation. Figure S6. Construction of dynamic temporal regulation cascade circuit fermentation strains. (a) PHB production followed by inhibition of TCA cycle. (b) Inhibition of TCA cycle followed by PHB production. (c) Inhibition of TCA cycle and production of PHB simultaneously. Figure S7. Growth of fermented strains. Figure S8. Glucose consumption of fermenting strains. Figure S9. The effect of CRISPRi system on gltA. Figure S10. Characterization of dynamic temporal control cascade circuit library. Figure S11. Glucose consumption of fermenting strains. (a) PHB production followed by inhibition of TCA cycle. (b) Inhibition of TCA cycle followed by PHB production. [file 13068_2023_2416_MOESM1_ESM.docx]

**Additional file1**

**Construction of Cascade** **Circuits for Dynamic Temporal Regulation and Its Application to PHB Production**

Xiaomeng Li^12^, Qingsheng Qi^1^ and Quanfeng Liang^1^*

1 State Key Laboratory of Microbial Technology, Shandong University, Qingdao, 266237, PR China

2 The Second Laboratory of Lanzhou Institute of Biological Products Co., Ltd, Lanzhou, 730046, PR China

* Correspondence: liangquanfeng@sdu.edu.cn

**1. Plasmid Construction**

**Construction of cascade circuit plasmids**

The base plasmids used to construct the QS module were 400-ecology, 213-gfp, and pBW313-LuxR-P_lux_-FDFP, which are maintained in our laboratory. PCR amplification was utilized to remove the *LasI* of plasmid 400-ecology and *TraI* of 213-gfp to obtain pBW400-LasR-P_las_-GFP and pBW213-TraR-P_tra*_-GFP, respectively. The reporter gene of plasmid pBW313-LuxR-P_lux_-FDFP was replaced with *gfp* to obtain pBW313-LuxR-P_lux_-GFP. On this basis, we used PCR and Gibson assembly to obtain plasmids for the exogenously induced expression of temporal regulatory circuits and self-induced temporal regulatory cascade circuits. The pBW213-TraR-P_tra*_-GFP plasmid was used as a template to ligate *lasR* to obtain pBW213-TraR-P_tra*_-LasR-GFP. Using pBW400-LasR-P_las_-GFP as a template, *lasR* was removed, and *gfp* was replaced with *rfp* to obtain P_las_-RFP. pBW213-TraR-P_tra*_-LasR-GFP was used as a template, and LasR was replaced to obtain pBW213-TraR-P_tra*_-LuxR-GFP.

pBW313-LuxR-P_lux_-GFP was used as a template, and *LuxR* was removed and replaced with *gfp* to obtain the plasmid P_lux_-RFP. Plasmid pBW400-LasR-P_tra*_-LasI-P_las_-RFP was utilized to amplify J23100 and *LasI*, which were ligated to pBW213-TraR-P_tra*_-LasR-GFP by Gibson assembly to obtain recombinant plasmid pBW213-LasI-TraR-P_tra*_-LasR-GFP. The QStet700 plasmid was used as a template to amplify the J23100 promoter, *luxI*, and Gibson assembled and transformed into the pBW213-TraR-P_tra*_-LasR-GFP plasmid vector to obtain pBW213-LuxI-TraR-P_tra*_-LuxR-GFP.

**Construction of time interval circuit library plasmids**

pBW213-LasI-TraR-P_tra*_-LasR-GFP was used as a plasmid construction template to construct a time interval circuit library. The promoter J23100 of LasI was replaced by J23102, J23104, J23101, J23106, J23110, J23114, J23113, J23112, and J23103; the promoter J23104 of TraR was replaced by J23100, J23102, J23106, J23110, J23114, J23113, J23112, and J23103; and the RBS30 of LasR was replaced by RBS31, RBS32, RBS33, RBS34, RBS64, RBS35, and RBS29 using PCR amplification and Gibson assembly. The plasmids L06-R32, T03-R35, L03-R32, L03-R29, L06-R33, L03-R33, T06-R35, T03-R34, and T06-R34 were obtained by combinatorial replacement (<http://parts.igem.org/Main_Page>).

**Construction of fermentation plasmids**

Using pBW213-P_tra*_-RFP as the template for plasmid construction, P_tra*_ was replaced with J23111, J23104, J23102, J23100, J23101, J23118, J23107, J23105, J23106, J23108, J23117, J23109, J23116, J23115, and J23114 according to the reverse PCR construction method to screen for a constitutive promoter of similar strength to the QS promoter for the construction of fermentation plasmids. Each fermentation strain included 3 plasmids. Plasmid QS-P_las_-dCas9 was obtained using pBW213-LasI-TraR-P_tra*_-LasR-GFP, pCDFTrc, and pCL-dCas9-CDA-UGI as templates. Plasmid P_las_-sgRNA was obtained using P_las_-RFP as a template. *Phbcab* was amplified using PBHR68 as a template, replacing the *rfp* in plasmid pBW213-P_tra*_-RFP to obtain P_tra*_-PHB. Then, QS-P_tra*_-dCas9, P_tra*_-sgRNA and P_las_-PHB, J23111-PHB, and J23102-PHB were obtained according to the method of reverse PCR.

**
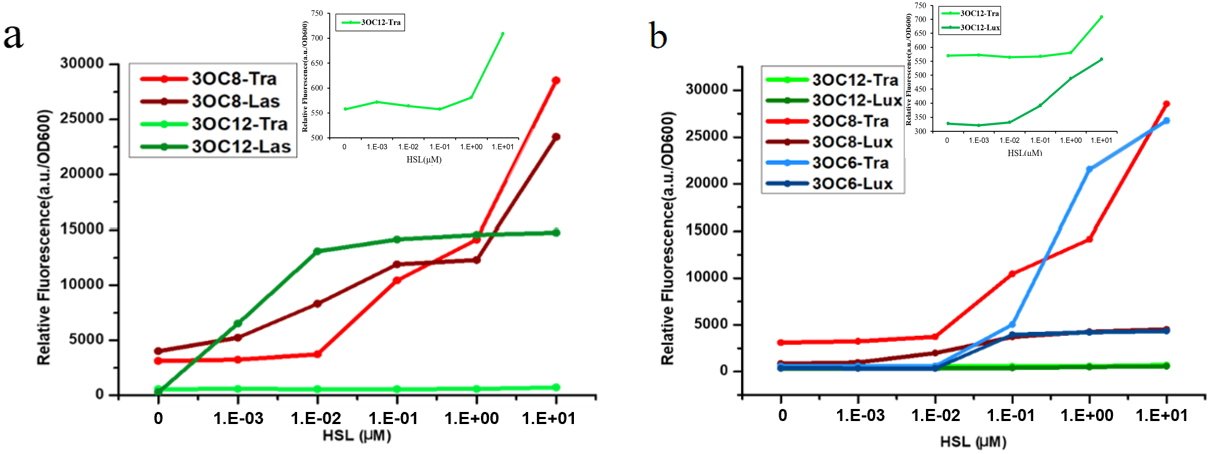
**

**Fig. S1** Verification of signal crosstalk in QS systems. (a) Characterization of signal crosstalk in Las and Tra systems. Add 3OC8HSL and 3OC12HSL in different concentrations (0, 1×10^-3^, 1×10^-2^, 1×10^-1^, 1, 10 µM), respectively. (b) Characterization of signal crosstalk in Lux and Tra systems. Add 3OC6HSL, 3OC8HSL and 3OC12HSL in different concentrations (0, 1×10^-3^, 1×10^-2^, 1×10^-1^, 1, 10 µM), respectively. The horizontal coordinate indicates the concentration of added HSL and the vertical coordinate indicates the fluorescence intensity/OD_600_= (fluorescence intensity of the experimental group - fluorescence intensity of the negative control group)/OD_600_.


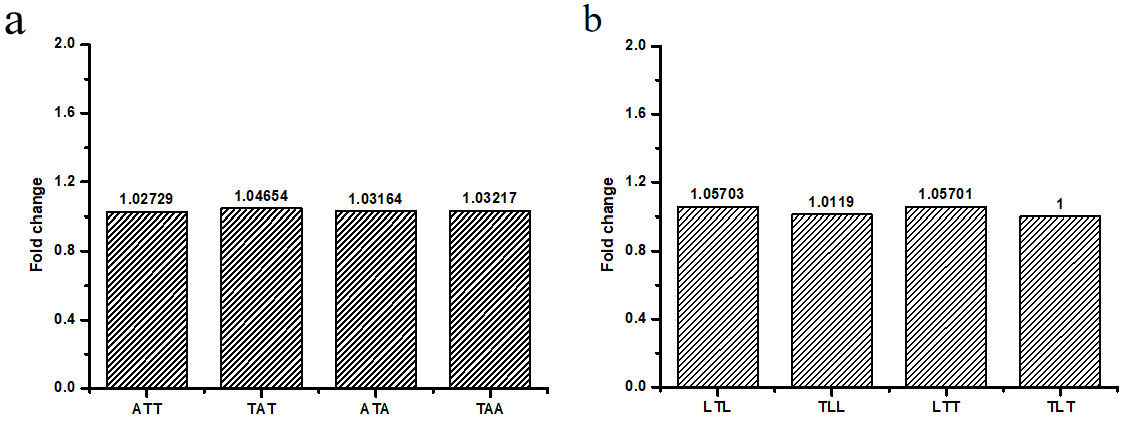


**Fig. S2** Verification of promoter orthogonality in QS systems. (a) Characterization of promoter orthologs in the Las, Tra system. Fold change in fluorescence intensity of two-plasmid strains when 100 μM concentration of HSL was added, ATT=P_las_+TraR+3OC8HSL, TAT=P_tra_+LasR+3OC8HSL, ATA=P_las_+TraR+3OC12HSL, TAA=P_tra_+LasR+3OC12HSL. (b) Characterization of promoter orthologs in the Lux, Tra system. Fold change in fluorescence intensity of two-plasmid strains when 100 μM concentration of HSL was added, LTL=P_lux_+TraR+3OC6HSL, TLL=P_tra_+LuxR+3OC6HSL, LTT=P_lux_+TraR+3OC8HSL, TLT=P_tra_+LuxR+3OC8HSL.

**
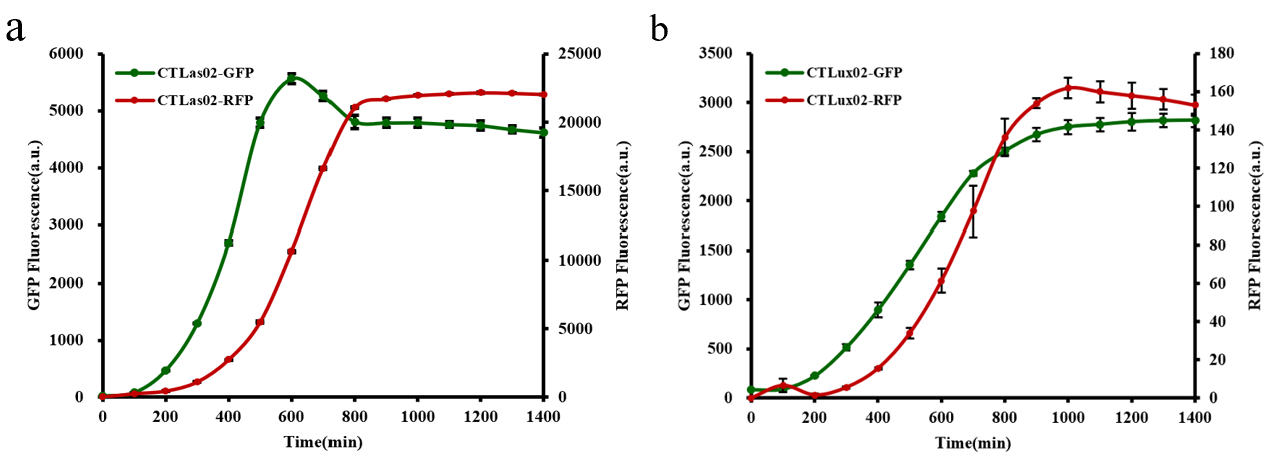
**

**Fig. S3** Construction of self-induced dynamic temporal regulation cascade circuits. (a) Characterization of self-induced dynamic temporal regulation cascade circuits based on Las, Tra systems. (b) Characterization of self-induced dynamic temporal regulation cascade circuits based on Lux, Tra systems.


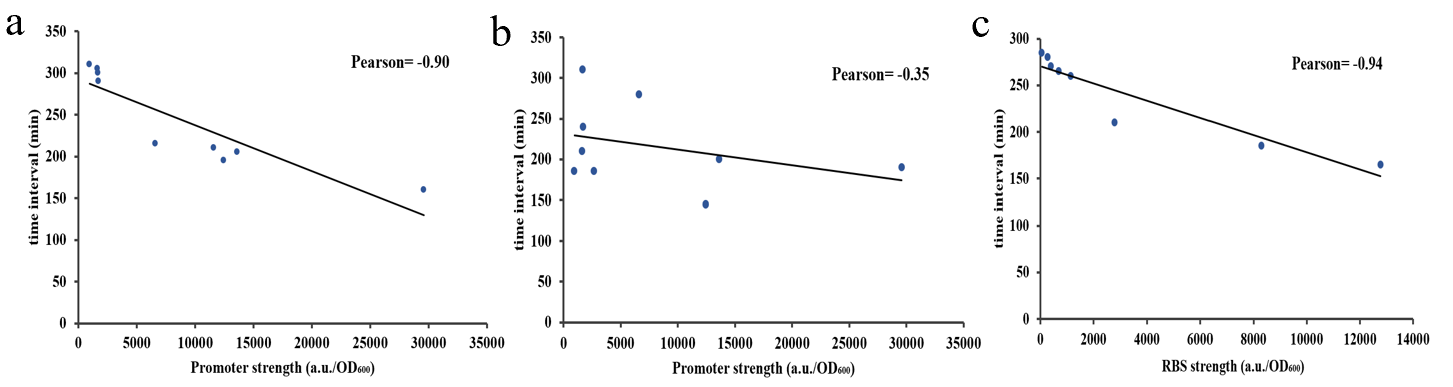


**Fig. S4** The rule between the time interval of the circuit and the strength of the promoter, RBS. (a) The relationship between promoter strength and the time interval obtained by regulating LasI. (b) The relationship between promoter strength and the time interval obtained by regulating TraR. (c) The relationship between RBS strength and the time interval obtained by regulating LasR (Pearson correlation coefficient is in the range of 0.8-1.0, and the relationship between X and Y is extremely strong; 0.6-0.8 indicates strong correlation; 0.4-0.6 indicates moderate correlation; 0.2-0.4 indicates weak correlation; 0-0.2 indicates very weak correlation or no correlation).


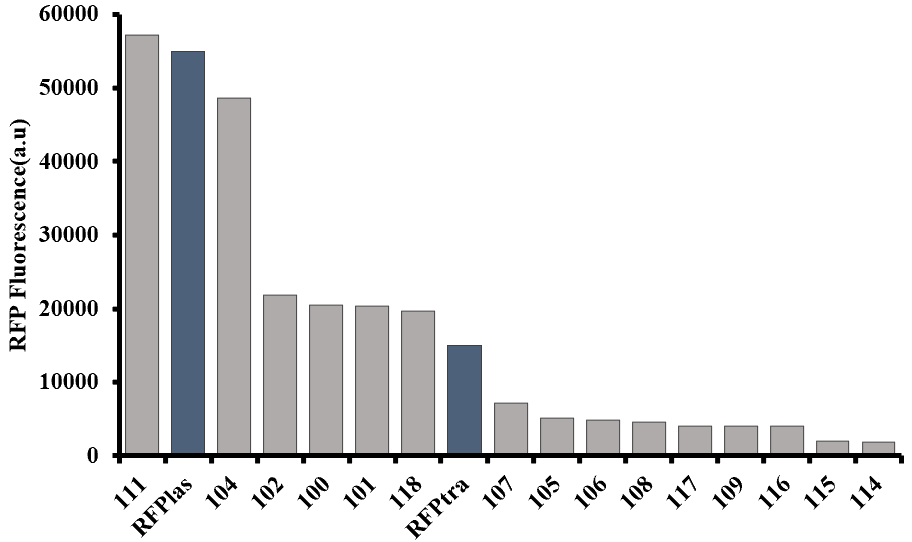


**Fig. S5** Screening of constitutive promoters in PHB fermentation.


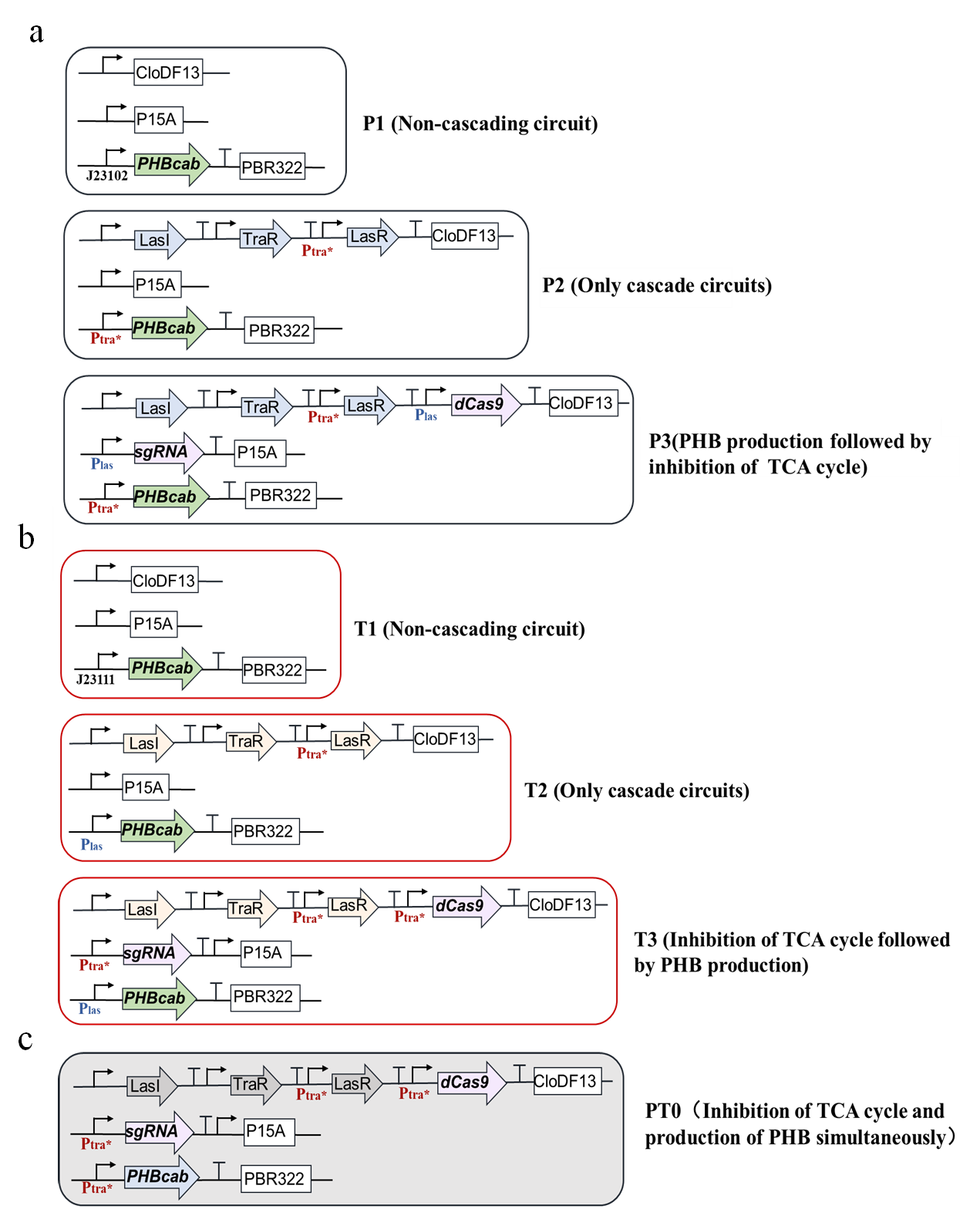


**Fig. S6** Construction of dynamic temporal regulation cascade circuit fermentation strains. (a) PHB production followed by inhibition of TCA cycle. (b) Inhibition of TCA cycle followed by PHB production. (c) Inhibition of TCA cycle and production of PHB simultaneously.

**
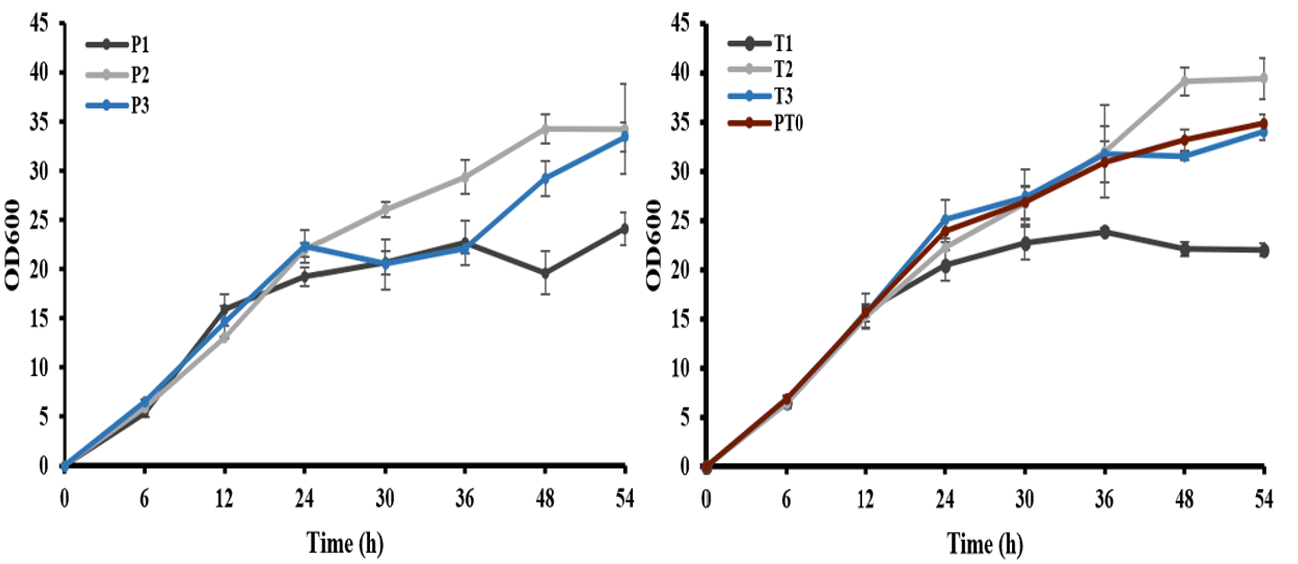
**

**Fig. S7** Growth of fermented strains.


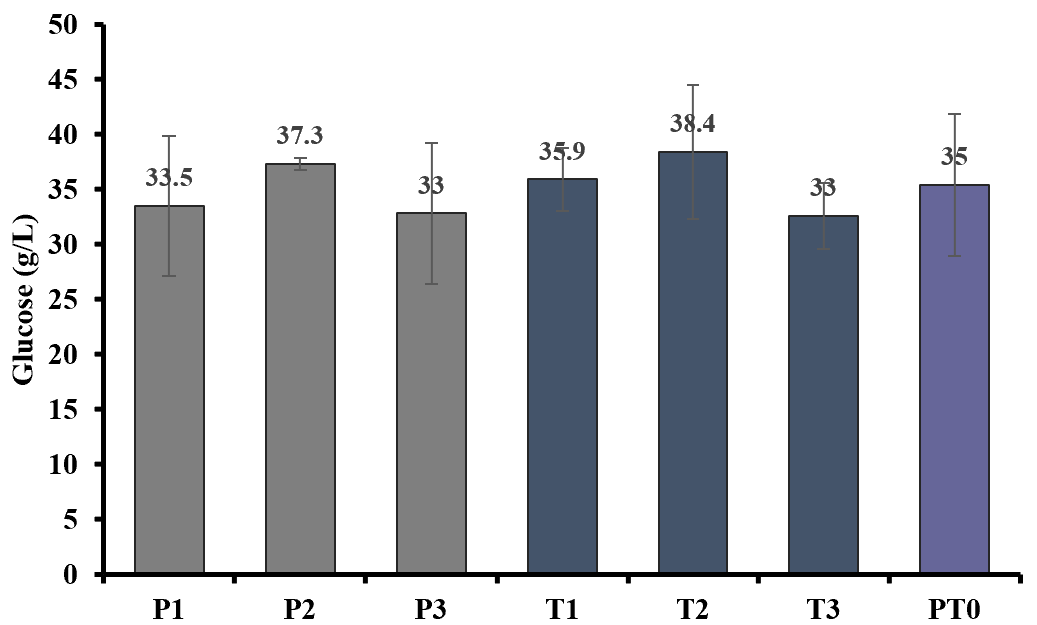


**Fig. S8** Glucose consumption of fermenting strains.


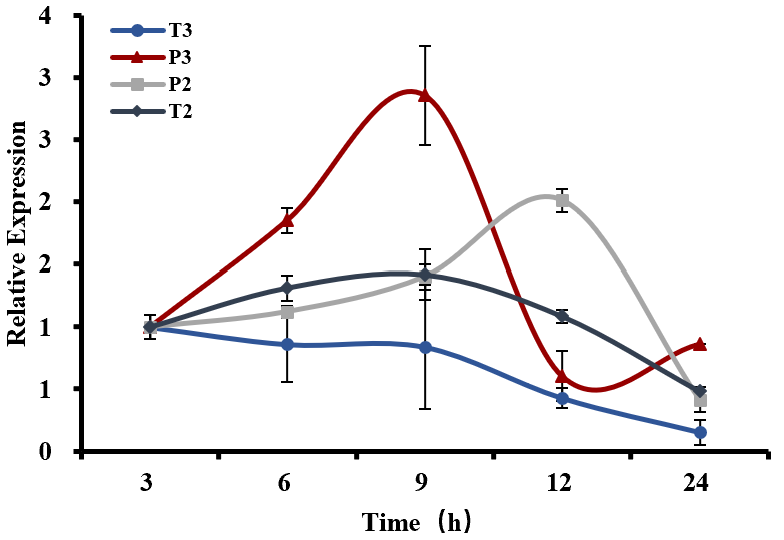


**Fig. S9** The effect of CRISPRi system on *gltA*. During the fermentation process, the experimental groups P3 and T3 and the control groups P2 and T2 were sampled at specific points, and RNA was extracted for real-time quantitative PCR.


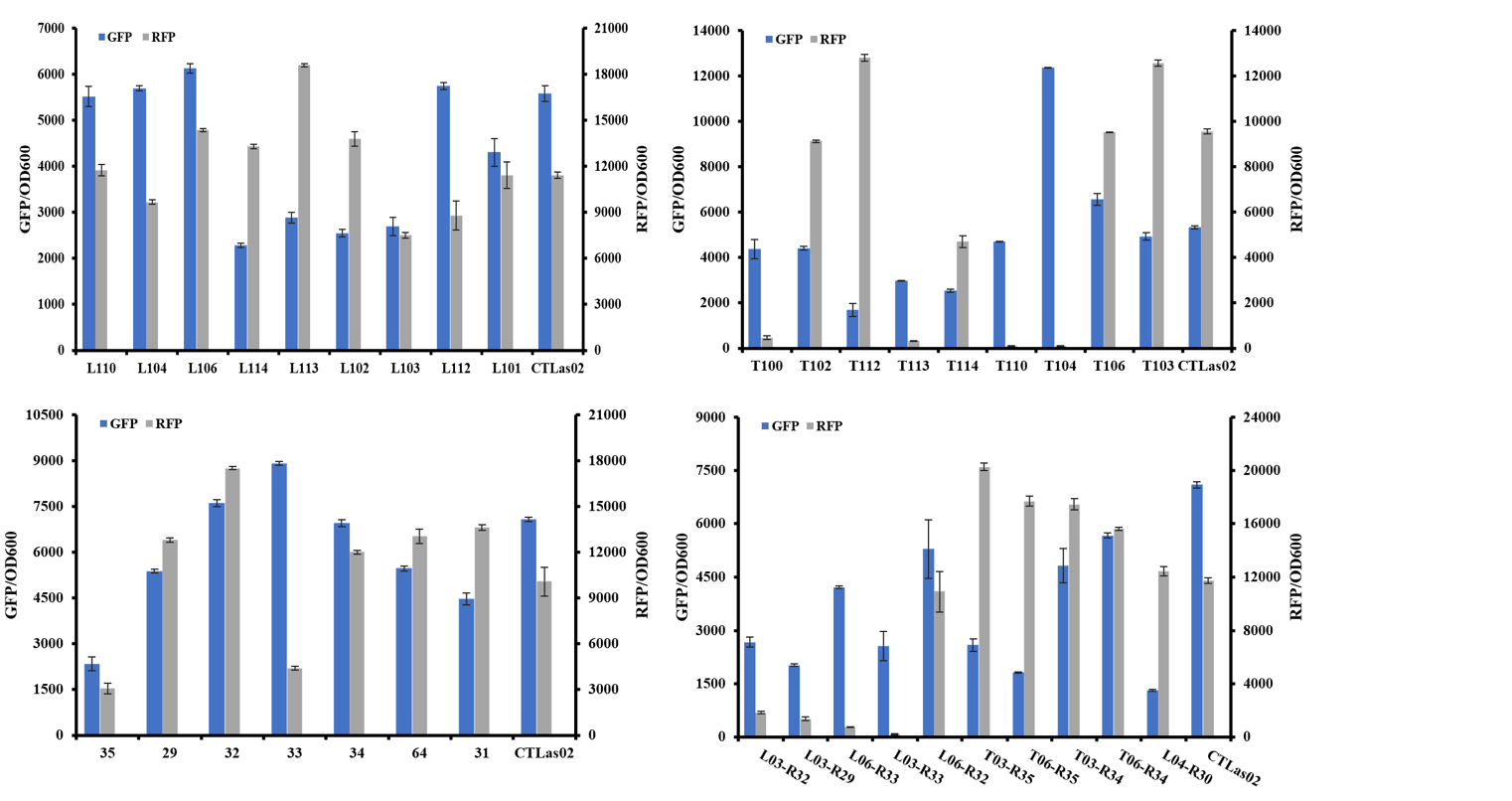


**Fig. S10** Characterization of dynamic temporal control cascade circuit library. The GFP and RFP fluorescence intensities of CTLas02 ranged from 5323 a.u.-7098 a.u. and 9561 a.u.-11719 a.u., respectively.


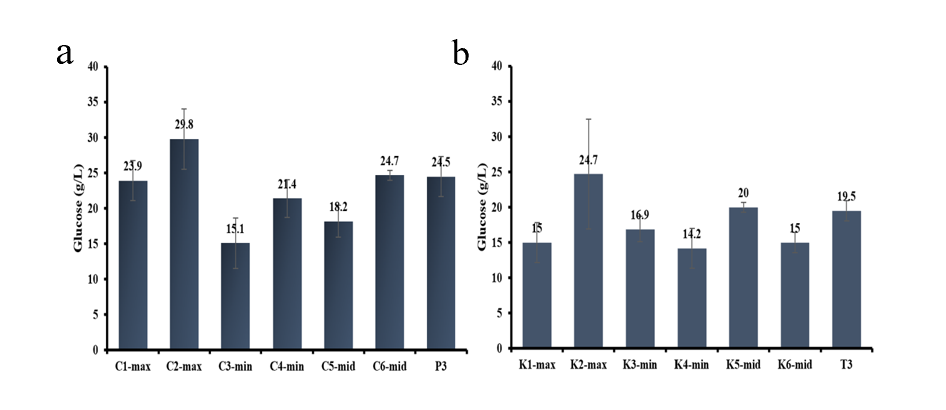


**Fig. S11** Glucose consumption of fermenting strains. (a) PHB production followed by inhibition of TCA cycle. (b) Inhibition of TCA cycle followed by PHB production.
